# MEETING PEOPLE WHERE THEY ARE: IMPLEMENTATION OF AN ACADEMIC DEMENTIA CARE MODEL IN A TEXAS HEALTH PLAN

**DOI:** 10.1093/geroni/igae098.1345

**Published:** 2024-12-31

**Authors:** Noemi Smithroat

**Affiliations:** Superior HealthPlan, Helotes, Texas, United States

## Abstract

Maximizing Independence at Home (MIND at Home), a well-researched holistic, family-centered dementia care coordination program, provides collaborative support to community-dwelling persons living with dementia (PLWD) and their informal care partners (CP). Through comprehensive home-based assessment of 13 memory-care domains covering PLWD and CPs, individualized care plans are created, implemented, monitored, and revised over the course of the illness. Non-clinical Memory Care Coordinators (MCCs) working with an interdisciplinary clinical team provide education and coaching to PLWD and their identified CP and serve as a critical liaison and resource navigators bridging families with medical professionals, and formal and informal community resources. This presentation will describe a pilot implementation of the program within Superior HealthPlan, a managed care organization, across diverse sites in Texas. We will present descriptions of how the program was effectively translated to practice, including the associated dementia support to enhance knowledge and skills, and review key aspects of the implementation experience including how the program was operationalized and adjustments made based on the challenges, obstacles, and opportunities; and summarize, the best practices that have emerged.

